# Japanese Alzheimer's Disease and Other Complex Disorders Diagnosis Based on Mitochondrial SNP Haplogroups

**DOI:** 10.1155/2012/245038

**Published:** 2012-07-17

**Authors:** Shigeru Takasaki

**Affiliations:** Toyo University, Izumino 1-1-1, Ora-gun Itakuracho, Gunma 374-0193, Japan

## Abstract

This paper first explains how the relations between Japanese Alzheimer's disease (AD) patients and their mitochondrial SNP frequencies at individual mtDNA positions examined using the radial basis function (RBF) network and a method based on RBF network predictions and that Japanese AD patients are associated with the haplogroups G2a and N9b1. It then describes a method for the initial diagnosis of Alzheimer's disease that is based on the mtSNP haplogroups of the AD patients. The method examines the relations between someone's mtDNA mutations and the mtSNPs of AD patients. As the mtSNP haplogroups thus obtained indicate which nucleotides of mtDNA loci are changed in the Alzheimer's patients, a person's probability of becoming an AD patient can be predicted by comparing those mtDNA mutations with that person's mtDNA mutations. The proposed method can also be used to diagnose diseases such as Parkinson's disease and type 2 diabetes and to identify people likely to become centenarians.

## 1. Introduction

Mitochondria are essential cytoplasmic organelles generating cellular energy in the form of adenosine triphosphate by oxidative phosphorylation. Because most cells contain hundreds of mitochondria, each having multiple copies of their mitochondrial DNA (mtDNA), each cell contains several thousand mtDNA copies. The mutation rate for mtDNA is very high, and when mtDNA mutations occur, the cells contain a mixture of wild-type and mutant mtDNAs. As the mutations accumulate, the percentage of mutant mtDNAs increases and the amount of energy produced within the cell can decline until it falls below the level necessary for the cell to function normally. When this bioenergetic threshold is crossed, disease symptoms appear and become progressively worse. Mitochondrial diseases encompass an extraordinary assemblage of clinical problems, usually involving tissues that require large amounts of energy, such as those in the heart, skeletal muscle, kidney, and endocrine glands [1–3]. 

Although there are reports that mtDNA mutations are related to aging and a wide variety of diseases—such as Alzheimer's disease (AD), Parkinson's disease (PD), type 2 diabetes (T2D) disease, and various kinds of cancer [4–20]—those reports focus on the amino acid replacements caused by mtDNA mutations. Mitochondrial functions can of course be affected directly by amino acid replacements, but they can also be affected indirectly by mutations in mtDNA control regions. It is, therefore, important to examine the relations between all mtDNA mutations and disease patients or centenarians.

In the work reported here, the relations between Japanese AD patients and their mitochondrial single nucleotide polymorphism (mtSNP) frequencies were first analyzed using a method based on radial basis function (RBF) networks [21, 22] and a method based on RBF network predictions [23]. The mtSNP haplogroups thus obtained were then used to predict whether or not someone will get Alzheimer's disease. It is also shown here that this diagnosis method based on the relations between PD patients, T2D patients, or centenarians and the mtSNPs of their haplogroups can also be used to diagnose other diseases and identify individuals likely to live a long time. The haplogroups described here are different from those reported previously [15, 16, 24, 25] and the proposed diagnosis method is the first one based on these haplogroups.

## 2. Materials and Methods

### 2.1. mtSNPs for Japanese People

Tanaka et al. sequenced the complete mitochondrial genomes of 672 Japanese individuals to construct an East Asia mitochondrial DNA (mtDNA) phylogeny [26]. Using these sequences and other published Asian sequences, they constructed the phylogenetic tree for macrohaplogroups M and N [26–28]. In the present study, the mtSNPs in various classes of people—96 Japanese Alzheimer's disease (AD) patients (20 males and 76 females, mean age: 77 ± 10 years; range 47 to 93 years), 96 Japanese Parkinson's disease (PD) patients (43 males and 53 females, mean age: 62 ± 9 years; range 39 to 81 years), 96 Japanese type 2 diabetes (T2D) patients (54 males and 42 females, mean age: 58 ± 5 years; range 43 to 65 years), 96 Japanese T2D patients with angiopathy (48 males and 48 females, mean age: 65 ± 10 years; range 43 to 92 years), 96 Japanese centenarians (30 males and 66 females, mean age: 100 ± 1 year; range 95 to 105 years), 96 Japanese healthy non-obese young males (96 males, mean age: 20 ± 3 years; range 18 to 25 years), and 96 Japanese healthy obese young males (96 males, mean age: 21 ± 2 years; range 18 to 25 years)—were obtained from the GiiB Human Mitochondrial Genome Polymorphism Database (http://mtsnp.tmig.or.jp/mtsnp), and the mtSNPs in 112 Japanese semi-supercentenarians (16 males and 96 females, mean age: 107.3 ± 1.2 years; range 105 to 115 years) were obtained from the report by Bilal et al. [25]. This paper, therefore, analyzed the mitochondrial genomes of 784 Japanese individuals although only 480 individuals were examined in Takasaki 2009 [23].

### 2.2. mtSNP Classification Using an RBF Network

The mtSNP classification for AD patients was examined using a radial basis function (RBF) and a method based on RBF network predictions. The RBF network is an artificial network used in supervised learning problems such as regression, classification, and time series prediction. In supervised learning, a function is inferred from examples (a training set) that a teacher supplies. The elements in the training set are paired values of the independent (input) variable and dependent (output) variable.

**Figure 1:**
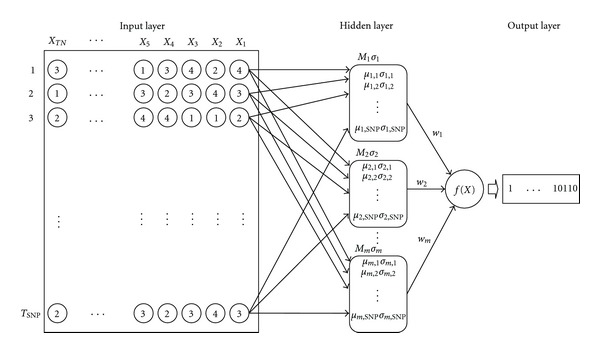
RBF network representation of the relations between individual mtSNPs and the AD patients. The input layer is the set of mtSNP sequences represented numerically (A, G, C, and T are converted to 1, 2, 3, and 4). The hidden layer classifies the input vectors into several clusters according to the similarities of individual input vectors. The determination of the output layer depends on which analysis is carried out. In the case of AD patients, 1 corresponds to AD patients and 0 corresponds to seven other classes of people. The other classes of people (PD patients, T2D patients, T2D patients with angiopathy, centenarians, semi-supercentenarians, non-obese young males, and obese young males) are carried out in a similar way. *X*_*i*_ is the *i*th input vector, TN is the maximum number of vectors (in this example, TN = 523 (64 × 7 + 75  (112 × 2/3)), *T*_SNP_ is the maximum number of mtSNPs (in this example, *T*_SNP_ = 562), *M*_*m*_ is the location vector, *m* is the number of basis functions, *μ* is the basis function, *σ* is the standard deviation, *w*_*i*_ is the *i*th weighting variable, and *f*(*X*) is the weighted sum function.

The RBF network shown in Figure 1 was learned from the training set as the mtSNPs of the AD patients were regarded as correct and the mtSNPs of other seven classes of people (i.e., PD patients, T2D patients, T2D patients with angiopathy, centenarians, semi-supercentenarians, obese young males, and non-obese young males) were regarded as incorrect. The mtSNP classifications for the other seven classes were carried out in the same way as that for the AD patients (Figure 1).

The mitochondrial genome sequences of the AD patients were partitioned into two sets: training data comprising the sequences of 64 of the 96 AD patients, and validation data comprising the sequences of the other 32 AD patients. The classification processes were carried out in two phases, training and validation, described in detail elsewhere [29].

### 2.3. Classification Based on Probabilities Predicted by the RBF Network

Since an RBF network can predict the probabilities that persons with certain mtSNPs belong to certain classes, these predicted probabilities were used to identify mtSNP features. Then other mtSNPs useful for distinguishing between the members in different classes were identified by examining the relations between individual mtSNPs and the persons with high predicted probabilities of belonging to one of these classes. Classification based on the probabilities predicted by the RBF network is carried out in the following way [23].

1. Select the target class to be analyzed.
2. Rank individuals according to their predicted probabilities of belonging to the target class.
3. Either select individuals whose probabilities are greater than a certain value or select the desired number of individuals and set them as a modified cluster.

### 2.4. Diagnosis of Various Diseases and Longevity

 As the proposed analysis method can predict a person's mtSNP constitution and probability of being an AD patient, PD patient, T2D patient, T2D patient with angiopathy, or a centenarian, it can be useful in the initial diagnoses of various diseases or longevity. The diagnosis can be checked in the following way.

1. Generate a table indicating the relations between mtDNA mutations and haplogroups of specified disease patients (e.g., AD patients, PD patients, T2D patients, T2D patients with angiopathy) or centenarians.
2. Examine the ratio of the mtDNA mutations of a certain person to the SNPs of the haplogroups for the specified disease patients or centenarians.
3. If the ratio is greater than a certain value (i.e., 0.8), the probability of that person's getting the specified disease or becoming a centenarian is higher than that of ordinary healthy persons.

Users can easily use the proposed method by using commercial or free RBF tools and Excel programs.

## 3. Results and Discussion

### 3.1. Associations between Japanese Haplogroups and mtSNPs of the AD Patients

**Figure 2:**
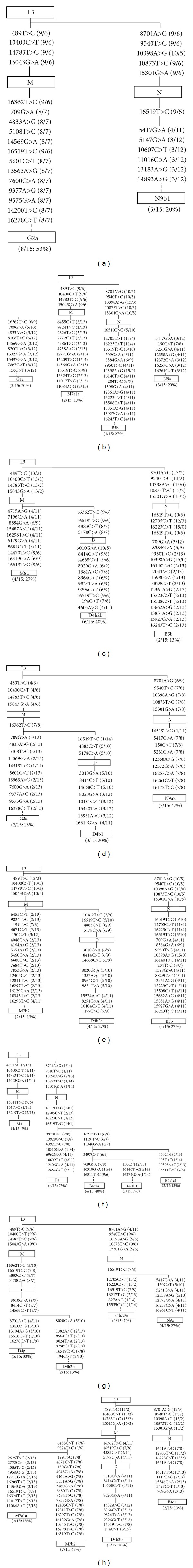
Associations between Japanese haplogroups and mtSNPs of the 15 individuals with the highest predicted probabilities. The description of the associations between Japanese haplogroups and mtSNPs is based on the phylogenetic tree for macrohaplogroups M and N described in Tanaka et al. [26]. The locus of mtDNA polymorphism (*mmm*), normal nucleotide (rCRS) at the position *mmm* (*N*_*N*_), mtDNA mutation at the same position (*N*_*M*_), the number of the mtDNA mutations at *mmm* in individual highest clusters (*Y*), and the number of the normal nucleotides at *mmm* in individual highest clusters (*X*) are expressed as *mmm N*_*N*_ > *N*_*M*_  (*Y*/*X*). In 16362 *T*>*C* (9/6), for example, 16362 is the mtDNA locus, T is the normal nucleotide at position 16362, C is the mtDNA mutation at that position, 9 is the number of mtDNA mutations, and 6 is the number of the normal nucleotides. (a) Japanese AD patients, (b) Japanese PD patients, (c) Japanese T2D patients, (d) Japanese T2D patients with angiopathy, (e) Japanese centenarians, (f) Japanese semi-supercentenarians, (g) Japanese non-obese young males, and (h) Japanese obese young males.

 When the mtSNPs of the AD patients were classified by the RBF-based method described above, eight mtSNP clusters were obtained. The average predicted probabilities of the people in these clusters becoming the AD patients are listed in Table 1. Since there were big differences among the predicted probabilities of 17 individuals in the cluster 1, the 15 individuals with the highest predicted probabilities of becoming AD patients were selected using the modified classification method, and their nucleotide distributions at individual mtDNA positions were examined. After that, the relations between Japanese haplogroups and the mtSNPs for the AD patients were examined [26–28]. The associations between the haplogroups and mtSNPs for the AD patients are shown in Figure 2(a). The features of associations for the AD patients were L3-M-G2a (53%) and L3-N-N9b1 (20%).

To compare the mitochondrial haplogroups of the AD patients with those of other classes of Japanese people, the relations between seven classes of Japanese people (Japanese PD patients, T2D patients, T2D patients with angiopathy, centenarians, semi-supercentenarians, non-obese young males, and obese young males) and their mtSNPs were also examined using the same modified method. The other seven associations between the haplogroups and mtSNPs for the PD patients, the T2D patients, the T2D patients with angiopathy, the centenarians, the semi-supercentenarians, the non-obese young males, and the obese young males are shown in Figures 2(b)–2(h). The relations among the haplogroups for all classes of people are listed in Table 2, from which it is clear that the haplogroups of the AD patients are different from those of other classes of Japanese people.

### 3.2. Alzheimer's Disease Diagnosis Based on the mtSNP Haplogroups

The relations between mtDNA mutations and the haplogroups of the AD patients shown in Figure 2(a) imply that the probability of becoming an AD patient is predicted by a person's mtSNP constitution. That is, if the haplogroups of a person are identified by examining his/her mtDNA mutations, that person's probability of becoming an AD patient might be also predicted by examining the relations between the mtDNA mutations and the mtSNPs of the haplogoups identified using the method described in Section 2. The relations between mtDNA loci and mtDNA mutations of the haplogroups G2a and N9b1 for the AD patients are listed in Table 3(a), and it is easy to check the relations between the mtDNA mutations and the mtSNPs of the haplogroups G2a and N9b1 by using that table. If, for example, someone's mtDNA mutations were A, G, C, T, A, G, A, G, A, C, and C at the loci 709, 4833, 5108, 5601, 7600, 9377, 9575, 13563, 14569, 16362, and 16519, one could see in Table 3(a) that those are all the mtDNA mutations of the haplogroup G2a except the ones at mtDNA positions 14200 and 16278. This implies that the person with those 11 mutations has a high probability of becoming an AD patient because the ratio of the mtDNA mutations to the SNPs of the haplogroup G2a is 0.84 (11/13).

This initial diagnosis method can be applied for other diseases or for the likelihood of longevity. The relations between the mtDNA mutations and haplogroups of the PD patients, T2D patients, T2D patients with angiopathy, centenarians, semi-supercentenarians, non-obese young males, and obese young males are listed in Tables 3(b)–3(h). In case of PD, one can see in Table 3(b) that if a person's mtDNA mutations were C, A, A, A, C, G, G, T, A, and C at the loci 204, 709, 1598, 8584, 9950, 12358, 12361, 15223, 15927, and 16140, that person would have a high probability of becoming a PD patient because the ratio of the mtDNA mutations to the SNPs of the haplogroup B5b is 0.83 (10/12). Similarly, one can see in Table 3(c) that if a person's mtDNA mutations were T, C, A, T, A, A, T, T, A, T, and C at loci 194, 1382, 3010, 4883, 5178, 8020, 8414, 8964, 9824, 14668, and 16519, that person would have high a probability of becoming a T2D patient because the ratio of the mtDNA mutations to the SNPs of the haplogroup D4b2b is 0.846 (11/13). The likelihood of longevity can also be diagnosed by the proposed method. One sees in Table 3(e) that if a person's mtDNA mutations were C, C, A, T, A, A, T, T, A, T, T, C, and C at the mtDNA loci 199, 1382, 3010, 4883, 5178, 8020, 8414, 8964, 9824, 10104, 14668, 16362, and 16519, that person would have a high probability of becoming a centenarian because the ratio of the mtDNA mutations to the SNPs of the haplogroup D4b2a is 0.867 (13/15).

### 3.3. Differences between Statistical Technique and the Modified RBF Method

Although the haplogroups of the AD patients were obtained by the modified RBF method, there are clear differences between the previously reported statistical technique and the method described here. The differences between standard statistical technique and the proposed method are listed in Table 4. In the statistical technique, the analysis of odds ratios or relative risks is based on the relative relations between target and control data at each polymorphic mtDNA locus. In the modified RBF method, on the other hand, clusters indicating predicted probabilities are examined on the basis of the RBF using correct and incorrect data for the entire polymorphic mtDNA loci. The statistical technique determines characteristics of haplogroups using independent mtDNA polymorphisms that indicate high odds ratios, whereas the modified RBF method determines them by checking individuals with high predicted probabilities. This means that the statistical technique uses the results of independent mutation positions, whereas the modified RBF method uses the results of entire mutation positions. As there are the differences between the two methods, which method is better depends on future research.

## 4. Conclusions

This paper examined the relations between Japanese AD patients and their mtSNPs by using the RBF network and a method based on RBF network predictions. As a result, Japanese AD patients were found to be associated with the haplogroups G2a and N9b1. Based on the mtSNPs of the haplogoups, a method for the initial diagnosis of Alzheimer's disease in Japanese people was proposed. The method can also be used to diagnose of other diseases and identify people likely to live a long time.

## Figures and Tables

**Table 1:** mtSNP classifications for the AD patients.

	Classification ID	Number of persons	Predicted probability (%)
AD patients	1	17	88.3
	2	33	45.6
	3	36	44.4
	4	27	40.7
	5	36	19.4
	6	39	0
	7	32	0
	8	303	0

PD patients	1	10	60
	2	16	56.3
	3	15	53.3
	4	17	41.2
	5	6	33.3
	6	13	30.8
	7	18	27.8
	8	21	19
	9	59	15.3
	10	17	11.8
	11	26	11.5
	12	10	10
	13	13	7.7
	14	24	4.2
	15	59	3.4
	16	9	0
	17	31	0
	18	9	0
	19	60	0
	20	6	0
	21	84	0

T2D patients	1	14	50
	2	48	35.4
	3	17	35.3
	4	31	29
	5	30	23.3
	6	40	17.5
	7	32	12.5
	8	36	8.3
	9	33	6.1
	10	47	4.3
	11	32	0
	12	89	0
	13	74	0

	1	16	81.3
T2D patients with angiopathy	2	19	57.9
	3	35	51.4
	4	39	43.6
	5	14	14.3
	6	32	9.4
	7	106	0
	8	262	0

Centenarians	1	39	66.7
	2	9	55.6
	3	26	42.3
	4	17	41.1
	5	15	40
	6	22	13.6
	7	23	13
	8	28	7.1
	9	20	5
	10	7	0
	11	22	0
	12	113	0
	13	85	0
	14	97	0

Semi-supercentenarians	1	12	66.7
	2	29	48.3
	3	48	35.4
	4	26	30.8
	5	30	20
	6	55	16.4
	7	47	14.9
	8	30	13.3
	9	24	4.2
	10	99	1
	11	24	0
	12	99	0

	1	28	57.1
	2	11	54.5
	3	12	50
	4	5	40
	5	16	37.5
	6	6	33.3
	7	23	21.7
Healthy non-obese young males	8	10	20
	9	62	17.7
	10	10	10
	11	12	8.3
	12	24	8.3
	13	27	7.4
	14	19	5.3
	15	29	3.4
	16	27	0
	17	15	0
	18	72	0
	19	115	0

Obese young males	1	32	62.5
	2	38	52.6
	3	29	31
	4	24	29.2
	5	28	28.6
	6	97	0
	7	275	0

**Table 2:** Haplogroup-class relations determined using the 15 individuals with highest probabilities.

			AD patients	PD patients	T2D patients	T2D patients with angiopathy	Centenarians	Semi-super- centenarians	Non-obese young males	Obese young males
M		M1						7%		
		M7a1a		13%						13%
		M7b2					13%			47%
		M8a			27%					
		G1a		20%						
		G2a	53%			13%				
	D	D4b1				20%				
		D4b2a					27%			
		D4b2b			40%				13%	20%
		D4g							33%	

		B4c1								13%
		B4c1a						40%		
		B4c1b1						7%		
		B4c1c1						13%		
		B4b/d/e							7%	
N		B4e								
		B5b		27%	13%		27%			
		F1						27%		
		N9a		20%					27%	
		N9a2				47%				
		N9b1	20%							

**Table:** (a)

mtDNA locus	Normal nucleotide	mtDNA mutation	AD patients	
			G2a	N9b1
709	G	A	A	
4833	A	G	G	
5108	T	C	C	
5147	G	A		A
5417	G	A		A
5601	C	T	T	
7600	G	A	A	
9377	A	G	G	
9575	G	A	A	
10607	C	T		T
11016	G	A		A
13183	A	G		G
13563	A	G	G	
14200	T	C	C	
14569	G	A	A	
14893	A	G		G
16278	C	T	T	
16362	T	C	C	
16519	T	C	C	

**Table:** (b)

mtDNA locus	Normal nucleotide	mtDNA mutation	PD patients			
			M7a1a	G1a	B5b	N9a
150	C	T		T		T
204	T	C			C	
709	G	A			A	
1598	G	A			A	
2626	T	C	C			
2772	C	T	T			
4386	T	C	C			
4958	A	G	G			
5231	G	A				A
5417	G	A				A
7867	C	T		T		
8020	G	A		A		
8584	G	A			A	
9950	T	C			C	
11017	T	C	C			
11084	A	G	G			
12358	A	G			G	
12361	A	G			G	
12372	G	A				A
12771	G	A	A			
14364	G	A	A			
15223	C	T			T	
15323	G	A		A		
15851	A	G			G	
15927	G	A			A	
16140	T	C			C	
16209	T	C		C		
16243	T	C			C	
16257	C	A				A
16261	C	T				T
16324	T	C	C			
16519	T	C	C			

**Table:** (c)

mtDNA locus	Normal nucleotide	mtDNA mutation	T2D patients		
			M8a	D4b2b	B5b
194	C	T		T	
204	T	C			C
709	G	A			A
1382	A	C		C	
1598	G	A			A
3010	G	A		A	
4715	A	G	G		
4883	C	T		T	
5178	C	A		A	
6179	G	A	A		
7196	C	A	A		
8020	G	A		A	
8414	C	T		T	
8584	G	A	A		A
8684	C	T	T		
8829	C	T			T
8964	C	T		T	
9296	C	T		T	
9824	T	A		A	
9950	T	C			C
12361	A	G			G
14470	T	C	C		
14605	A	G		G	
14668	C	T		T	
15223	C	T			T
15487	A	T	T		
15508	C	T			T
15662	A	G			G
15851	A	G			G
15927	G	A			A
16140	T	C			C
16243	T	C			C
16298	T	C	C		
16319	G	A	A		
16519	T	C	C	C	

**Table:** (d)

mtDNA locus	Normal nucleotide	mtDNA mutation	T2D patients with angiopathy		
			G2a	D4b1	N9a2
150	C	T			T
709	G	A	A		
3010	G	A		A	
4833	A	G	G		
4883	C	T		T	
5108	T	C	C		
5178	C	A		A	
5231	G	A			A
5417	G	A			A
5601	C	T	T		
7600	G	A	A		
8020	G	A		A	
8414	C	T		T	
9377	A	G	G		
9575	G	A	A		
10181	C	T		T	
12358	A	G			G
12372	G	A			A
13563	A	G	G		
14569	G	A	A		
14668	C	T		T	
15440	T	C		C	
15951	A	G		G	
16172	T	C			C
16257	C	A			A
16261	C	T			T
16278	C	T	T		
16319	G	A		A	
16362	T	C	C	C	
16519	T	C	C	C	C

**Table:** (e)

mtDNA locus	Normal nucleotide	mtDNA mutation	Centenarians		
			M7b2	D4b2a	B5b
150	C	T	T		
199	T	C		C	
204	T	C	C		
709	G	A			A
1382	A	C		C	
1598	G	A			A
3010	G	A		A	
4048	G	A	A		
4071	C	T	T		
4164	A	G	G		
4883	C	T		T	
5178	C	A		A	
5351	A	G	G		
5460	G	A	A		
6455	C	T	T		
6680	T	C	C		
7684	T	C	C		
7853	G	A	A		
8020	G	A		A	
8251	G	A		A	
8414	C	T		T	
8584	G	A			A
8829	C	T			T
8964	C	T		T	
9824	T	A		A	
9824	T	C		C	
9950	T	C			C
10104	C	T		T	
10345	T	C	C		
12361	A	G			G
12405	C	T	T		
12705	C	T			T
12811	T	C	C		
14668	C	T		T	
15223	C	T			T
15508	C	T			T
15662	A	G			G
15851	A	G			G
15927	G	A			A
16129	G	A	A		
16140	T	C			C
16223	C	T			T
16243	T	C			C
16297	T	C	C		
16298	T	C	C		
16362	T	C		C	
16519	T	C		C	C

**Table:** (f)

mtDNA locus	Normal nucleotide	mtDNA mutation	Semi-supercentenarians				
			M1	B4c1a	B4c1b1	B4c1c1	F1
150	C	T			T	T	
195	T	C	C			C	
709	G	A		A			
1119	T	C		C	C	C	
1621	T	C		C	C	C	
3497	C	T		T	T		
3970	C	T					T
6392	T	C					C
6962	G	A					A
10310	G	A		A			A
10398	A	G				G	
10609	G	A					A
12406	G	A					A
12705	C	T		T	T	T	T
12802	C	T					T
13928	G	C					C
15346	G	A		A			
16140	T	C			C		
16217	T	C		C	C	C	
16223	C	T		T	T	T	T
16249	T	C	C				
16274	G	A			A		
16311	T	C	C	C			
16519	T	C		C	C	C	C

**Table:** (g)

mtDNA locus	Normal nucleotide	mtDNA mutation	Non-obese young males			
			D4b2b	D4g	B4b/d/e	N9a
150	C	T				T
194	C	T	T			
827	A	G			G	
1382	A	C	C			
3010	G	A		A		
4343	A	G		G		
4883	C	T	T	T		
5178	C	A	A	A		
5231	G	A				A
5417	G	A				A
8020	G	A	A			
8414	C	T		T		
8701	A	G		G		
8964	C	T	T			
9296	C	T	T			
9824	T	A	A			
12358	A	G				G
12372	G	A				A
12705	C	T			T	
13104	A	G		G		
14668	C	T	T	T		
15518	C	T		T		
15535	C	T			T	
16217	T	C			C	
16223	C	T			T	
16257	C	A				A
16261	C	T				T
16278	C	T		T		
16362	T	C	C	C		
16519	T	C	C	C	C	C

**Table:** (h)

			Obese young males			
mtDNA locus	Normal nucleotide	mtDNA mutation	M7a1a	M7b2	D4b2b	B4c1
150	C	T		T		
194	C	T			T	
199	T	C		C		
709	G	A				A
1119	T	C				C
1382	A	C			C	
2626	T	C	C			
2772	C	T	T			
3010	G	A			A	
3497	C	T				T
4048	G	A		A		
4071	C	T		T		
4164	A	G		G		
4386	T	C	C			
4883	C	T			T	
4958	A	G	G			
5178	C	A			A	
5351	A	G		G		
5460	G	A		A		
6455	C	T	T	T		
6680	T	C		C		
7684	T	C		C		
7853	G	A		A		
8020	G	A			A	
8964	C	T			T	
9296	C	T			T	
9824	T	A			A	
10345	T	C		C		
12405	C	T		T		
12705	C	T				T
12771	G	A	A			
12811	T	C		C		
14668	C	T			T	
15346	G	A				A
16129	G	A		A		
16209	T	C	C			
16217	T	C				C
16223	C	T				T
16297	T	C		C		
16298	T	C		C		
16324	T	C			C	
16362	T	C			C	
16519	T	C		C	C	

**Table 4:** Differences between the statistical technique and the proposed (modified RBF) method.

	Statistical technique	Proposed method
Technique	Relative relations between target and normal data	Supervised learning (RBF) by using correct and incorrect data
Analysis position	Each locus of mtDNA polymorphisms (independent position)	Entire loci of mtDNA polymorphisms (succesive positions)
Input (required data)	Target (individual cases) and control (normal data)	Correct (individual cases) and incorrect (others except correct)
Output (results)	Odds ratio or relative risk	Clusters with predictions
Analysis	Check odds ratio or relative risk at each position	Check individuals in clusters based on prediction probabilities
